# Enjoyment, anxiety, and boredom in Spanish learning: a study of Chinese university students

**DOI:** 10.3389/fpsyg.2025.1616036

**Published:** 2026-01-06

**Authors:** Nan Zhou, Yitong Liu

**Affiliations:** Renmin University of China, Beijing, China

**Keywords:** Chinese learners, cultural psychology, foreign language emotions, language education, Spanish learning

## Abstract

Grounded in the Control–Value Theory of Achievement Emotions and positive psychology perspectives, this study explores how emotions influence Spanish language learning among Chinese university students, focusing on enjoyment, anxiety, and boredom in classroom settings. Using a structured questionnaire, data were collected from 158 Spanish majors at four Chinese universities. Emotional variables and cultural-psychological factors were analyzed through regression and correlation analysis. Cultural influences (e.g., face-saving, high-context communication) were examined through embedded survey items and their statistical associations with emotional variables. Findings show that enjoyment is positively correlated with self-perceived effectiveness, while anxiety was negatively associated with exam performance. Boredom is linked to disengagement and lower self-efficacy. Emotional patterns were significantly influenced by cultural traits such as face-saving, high-context communication, and exam orientation. Given the convenience sample of 158 students from four universities, findings should be interpreted with caution regarding generalizability. The results highlight the need for culturally responsive pedagogy in foreign language education. Implications include integrating emotion-focused instructional practices, promoting psychologically safe classroom environments, and adapting teaching strategies to culturally shaped communication styles.

## 1 Introduction

In recent years, emotional factors have become critical in foreign language education, influencing not only motivation and classroom performance but also learners’ overall engagement and outcomes. In Chinese university Spanish programs, students face both the cognitive challenges of learning a less commonly taught language and the psychological effects of cultural expectations, educational pressures, and individual learning orientations.

While research has explored emotional dynamics in English learning, studies on Spanish—especially within the Chinese context—remain limited. Emotions such as enjoyment, anxiety, and boredom often co-occur in language learning and significantly impact performance and self-perception (Dewaele and MacIntyre, 2014; Dewaele et al., 2023). However, the specific emotional patterns in Spanish learning, particularly among Chinese learners, have not been thoroughly explored. Notably, MacIntyre and Gregersen (2012) have advocated a positive psychology approach in Second Language Acquisition, emphasizing that cultivating positive emotions can broaden learners’ capacity to overcome challenges—a reminder that both positive and negative emotions must be examined critically rather than in isolation.

Spanish, unlike English, lacks the same broad societal utility in China, leading to what can be described as “motivational drift.” Spanish learners have fewer opportunities for real-world practice and cultural integration, which contributes to emotional fatigue. Additionally, Chinese educational traditions prioritize accuracy over fluency, which hinders spontaneous speech and emotional engagement, unlike English learners who have more opportunities for authentic language use outside the classroom.

This study investigates three core emotions—Foreign Language Enjoyment (FLE), Foreign Language Anxiety (FLA), and Foreign Language Boredom (FLB)—among Chinese university students studying Spanish. It explores how these emotions interact and how they predict both subjective and objective learning outcomes. The study also examines how cultural-psychological traits, such as face-saving, high-context communication, and exam-oriented learning, shape students’ emotional experiences.

Using a quantitative design based on the Español Moderno course, the study integrates surveys and regression analyses to model the relationships between emotion, cultural context, and language performance.

Prior research has shown that culturally shaped psychological traits—such as face-saving concerns, high-context communication styles, and collectivist orientations—strongly influence learners’ emotional responses and classroom behaviors (Hu, 1944; Hall, 1976; Wen and Clément, 2003; Teimouri et al., 2019). These culturally embedded factors may interact with enjoyment, anxiety, and boredom, thereby shaping learning outcomes. This provides the conceptual justification for examining cultural-psychological variables in RQ3.

The study aims to address the following research questions:

- RQ1: How do FLE, FLA, and FLB interact in the context of Chinese Spanish language classrooms?
- RQ2: To what extent do FLE, FLA, and FLB predict students’ learning outcomes, including both objective performance (exam scores) and subjective perceptions of learning effectiveness?
- RQ3: How are these emotional experiences shaped by cultural-psychological factors such as face-saving concerns, high-context communication, and exam-oriented learning?

## 2 Theoretical framework and empirical foundations

This study is primarily guided by the Control–Value Theory of Achievement Emotions (CVT; Pekrun, 2006), with the Broaden-and-Build Theory (BBT; Fredrickson, 2001, 2003) serving as a supplement. CVT explains how enjoyment, anxiety, and boredom stem from learners’ control–value appraisals and affect learning outcomes, while BBT highlights the long-term benefits of enjoyment in broadening engagement and building lasting resources. Together, these frameworks clarify how emotions shape learning and provide a conceptual basis for research in L2/L3 acquisition, including less commonly taught languages (Wu and Kabilan, 2025). It should be noted that in this study these emotions are conceptualized as state-like achievement emotions specific to the Spanish classroom context (i.e., situational feelings at the time of data collection), rather than stable trait dispositions.

### 2.1 Control–Value Theory of Achievement Emotions

CVT conceptualizes emotions as consequences of learners’ appraisals of two factors: perceived control over learning activities and perceived value of those activities (Pekrun, 2006). Positive emotions such as enjoyment or pride occur when students believe they can manage learning tasks and consider them worthwhile; negative emotions such as anxiety or boredom arise when control is low or when the task is perceived as meaningless. In this framework, factors such as face-saving and exam pressure influence perceived control and task value, aligning with CVT’s explanation of how emotional appraisals shape achievement emotions.

Research grounded in Control–Value Theory shows that Chinese university students’ positive emotions—such as enjoyment, hope, and pride—tend to arise when learners perceive strong control over and value in language-learning tasks, whereas boredom and anxiety generally emerge under low-control or low-value appraisals (Shao et al., 2020). In addition, empirical work confirms that enjoyment and anxiety function as distinct yet coexisting emotions in the foreign language classroom, each exerting a significant influence on learners’ willingness to communicate (Dewaele and MacIntyre, 2014). More recent studies further confirm the applicability of CVT by showing how classroom structures and assessment systems shape learners’ emotional responses (Dewaele et al., 2023).

### 2.2 Broaden-and-build theory of positive emotions

Fredrickson’s Broaden-and-Build Theory (2001, 2003) emphasizes the functional advantages of positive emotions. Positive emotions, such as enjoyment, expand learners’ thought–action repertoires, encouraging creativity, exploration, and risk-taking. Over time, they help build lasting cognitive, motivational, and social resources, including resilience and self-confidence.

Empirical work in foreign language education supports these claims. Evidence indicates that foreign language enjoyment can enhance learners’ persistence and capacity for self-regulated learning (Li et al., 2018). Subsequent research further shows that teacher behaviors—particularly warmth, humor, and constructive feedback—serve as strong predictors of classroom enjoyment, which in turn contributes to higher motivation and improved performance (Dewaele et al., 2023). Structural modeling from this line of work also suggests that increases in enjoyment do not necessarily translate into reductions in anxiety or boredom, indicating that positive emotions and negative emotions may operate through partially independent mechanisms. By contrast, negative emotions function differently: anxiety narrows attention, hinders communication, and reduces participation (Dong et al., 2022; Horwitz, 2001), while boredom undermines engagement and contributes to academic burnout (Pekrun et al., 2010; Zhang, 2022). In this study, BBT is used as a secondary framework to accentuate the long-term broadening and resource-building role of enjoyment, thereby complementing CVT’s focus on the immediate antecedents of achievement emotions.

### 2.3 Enjoyment, anxiety, and boredom in language learning

Among the wide spectrum of academic emotions, FLE, FLA, and FLB have been most consistently examined in foreign language learning research.

- FLE is associated with learners’ sense of achievement, positive classroom interactions, and intrinsic or extrinsic motivation. Interactive and collaborative classroom activities foster greater enjoyment, which in turn sustains motivation (Dewaele et al., 2023; Dewaele and MacIntyre, 2014).
- FLA remains a key barrier to learning. It typically manifests as communication apprehension, test-related stress, and fear of negative evaluation (Horwitz, 2001). In Chinese contexts, cultural emphasis on accuracy and face-saving often amplifies such anxiety, particularly in speaking tasks (Dong et al., 2022).
- FLB has recently been recognized as a distinct and consequential emotion in education. It is linked to monotonous tasks, low task relevance, and repetitive pedagogy. Research indicates that boredom undermines attention and participation, while task variety and cultural integration help mitigate it (Pekrun et al., 2010; Zhang, 2022).

Interplay among FLE, FLA, and FLB is increasingly emphasized in recent scholarship. Rather than being isolated constructs, these emotions often co-occur and dynamically interact to shape learners’ willingness to communicate, their motivation, and their academic resilience (Wu and Kabilan, 2025).

### 2.4 Implications for Spanish language learning in China

Building on the theoretical foundations of CVT and Broaden-and-Build Theory, and the empirical findings across L2/L3 contexts, the following subsection highlights why Spanish, as a less commonly taught language in China, presents distinctive emotional patterns. While much empirical research has focused on English as an L2, these theoretical frameworks and findings apply equally to Spanish as an L2 or L3. In China, Spanish is typically introduced at university, where learners’ motivations may be rooted in cultural interest or career opportunities rather than standardized testing. This context amplifies the influence of classroom climate and instructional practices on emotional outcomes. Enjoyment is particularly important in sustaining engagement, while anxiety and boredom reflect the tension between exam-oriented educational traditions and the communicative demands of language learning.

Unlike English, which enjoys widespread social use, abundant resources, and a firmly institutionalized testing system in China, Spanish is introduced later and lacks comparable environmental support. As a result, learners’ emotions are shaped more directly by classroom dynamics and cultural distance. Prior research shows that while English learners often report anxiety tied to standardized testing and social expectations (Dong et al., 2022; Horwitz, 2001), Spanish learners experience anxiety more strongly due to limited opportunities for authentic communication and the fragility of their learning environment (Wen and Clément, 2003; Yu, 2016). Similarly, enjoyment in English classrooms often benefits from extracurricular exposure, whereas in Spanish learning it depends more heavily on teacher behaviors and classroom interaction (Dewaele et al., 2023). By highlighting these contrasts, the present study contributes to filling a gap in the literature, offering insights into how established theories of emotion apply in the less examined but increasingly important context of Spanish as an L2 or L3. This highlights that Spanish learning in China offers a distinctive testing ground for theories of academic emotions, complementing but also diverging from the well-documented English L2 context.

### 2.5 Emotional dynamics in Spanish vs. other foreign languages in China

Emotional dynamics in learning Spanish share similarities with those in learning other foreign languages, such as English, but Spanish as an L2/L3 in China presents unique challenges. Unlike English, which has broad societal and professional utility, Spanish lacks the same external reinforcement, leading to “motivational drift.” Fewer opportunities for real-world use and cultural integration contribute to emotional fatigue among Spanish learners.

Additionally, Chinese educational traditions emphasize accuracy over fluency, which conflicts with the trial-and-error nature of language learning. Chinese students tend to be more risk-averse, prioritizing correctness over fluency (Biggs, 1996; Hu, 1944), which creates emotional barriers and reduces their willingness to engage in spontaneous speech. In contrast, English learners benefit from more exposure to the language outside the classroom, normalizing mistakes as part of the learning process.

Moreover, Chinese learners’ sensitivity to peer judgment exacerbates anxiety in small-group Spanish classes. Fear of making mistakes leads to avoidance behaviors, hindering classroom participation. Teachers who rely on traditional methods without fostering a safe, communicative environment contribute to this issue.

Given these challenges, Spanish instruction in China should focus on creating a classroom environment that promotes psychological safety, where mistakes are viewed as opportunities for growth. Moving away from rigid, exam-driven models and emphasizing communicative competence will help maintain emotional engagement and enhance both language proficiency and emotional well-being.

## 3 Relationship between emotion and learning outcomes

A substantial body of research in educational psychology and applied linguistics demonstrates that emotions play a decisive role in shaping learning processes and outcomes (Pekrun, 2006; Fredrickson, 2001; Dewaele and MacIntyre, 2014). Emotions influence motivation, attention, self-regulation, and persistence, thereby directly affecting achievement. In second language acquisition, where learners constantly face challenges of communication and self-expression, emotions are especially salient. This section reviews the general relationship between emotions and learning outcomes, with a focus on three central dimensions: positive emotions, negative emotions, and boredom.

### 3.1 Positive emotions and learning gains

Positive emotions such as enjoyment, interest, hope, and pride have consistently been shown to facilitate learning. According to the Broaden-and-Build Theory (Fredrickson, 2001, 2003), positive emotions expand learners’ thought–action repertoires, fostering creativity, flexibility, and resilience. In language learning, enjoyment is particularly influential: it increases willingness to communicate, strengthens persistence, and promotes the use of deeper learning strategies (Dewaele and MacIntyre, 2014; Li et al., 2018).

Empirical studies confirm these patterns. Pekrun and Perry (2014) reported that enjoyment and pride were positively associated with achievement across academic domains, while Shao et al. (2020) found similar results in foreign language classrooms in China. Learners who experienced enjoyment and interest invested more effort, demonstrated greater engagement, and achieved higher performance. These findings underscore the constructive role of positive emotions in second language acquisition.

### 3.2 Negative emotions and learning barriers

In contrast, negative emotions can significantly hinder learning. Foreign Language Anxiety (FLA), encompassing communication apprehension, test anxiety, and fear of negative evaluation, has been widely documented as a barrier to effective language acquisition (Horwitz, 2001; MacIntyre and Gregersen, 2012). Anxiety narrows attentional focus, reduces working memory capacity, and discourages risk-taking behaviors essential to language practice. Learners experiencing high levels of anxiety often remain silent in class, avoid participation, and underperform in assessments (Dong et al., 2022; Liu and Jackson, 2008).

Test anxiety, in particular, has been linked to lower achievement and greater emotional exhaustion. Studies indicate that anxious learners are more likely to adopt avoidance strategies, blame themselves for difficulties, and withdraw from active learning (Cheng and Zhang, 2025). These patterns show that anxiety not only impedes immediate performance but also erodes long-term motivation.

### 3.3 Boredom and academic disengagement

Boredom, though less studied than anxiety, has emerged as a distinct negative emotion with substantial consequences for learning outcomes. It is characterized by inattention, lack of challenge, and disengagement from classroom activities. Pekrun et al. (2010) highlighted boredom as a critical factor undermining both motivation and performance, while Dewaele and Li (2021) showed that boredom negatively correlates with academic achievement in language learning.

Classroom boredom often arises from repetitive tasks, low contextual relevance, or teacher-centered instruction (Zhang, 2022). In such environments, learners disengage psychologically, reducing both cognitive investment and communicative output. Consequently, boredom represents not merely the absence of enjoyment but an independent impediment to learning.

### 3.4 Gaps in Spanish language learning research

Although research on emotions in language learning has made significant progress, much of the literature has focused on English as a second language (L2). Research specifically addressing the emotional dynamics in Spanish as a second or third language (L2/L3) is limited, particularly in the Chinese context. While theories like CVT and the Broaden-and-Build Theory have been applied widely in L2 studies, there remains a gap in understanding how these theories apply to less commonly taught languages like Spanish.

Spanish learning in China presents unique challenges, including limited institutional support and fewer opportunities for real-world language use compared to English. These contextual differences lead to distinct emotional experiences for learners, shaped by classroom practices and cultural factors. Thus, while much of the emotional research has focused on English language learners, the present study aims to address this gap by exploring how emotions like enjoyment, anxiety, and boredom operate in the context of Spanish language learning, offering valuable insights for educators and researchers in this underexplored area.

## 4 Psychological characteristics of Chinese learners in Spanish language learning

Learning Spanish among Chinese university students is an emotionally and culturally embedded process. Learners’ experiences are influenced by cultural factors, the exam-oriented educational system, and societal as well as familial expectations. These psychological forces shape learners’ emotional responses—especially enjoyment, anxiety, and boredom—which significantly affect language acquisition.

### 4.1 Cultural influences on learning emotions

Cultural factors strongly determine Chinese learners’ affective experiences in Spanish classrooms. The concept of face plays a central role in Chinese society (Hu, 1944), leading students to worry about losing dignity when making mistakes in pronunciation or grammar. This fear of “losing face” contributes to high levels of language anxiety and a tendency to remain silent (Cheng and Zhang, 2025; Yu, 2016). Additionally, China’s communication style has been classified as high-context (Hall, 1976), emphasizing indirectness and non-verbal cues. This contrasts with the low-context, direct, and spontaneous nature of Spanish, creating tension for Chinese learners who often delay speech or avoid participation (Wen and Clément, 2003). Moreover, collectivist values heighten sensitivity to peer evaluation, as errors in class may be perceived as disrupting group harmony, reinforcing hesitation and passivity in interactions (Rui and Ji, 2017; Wen and Clément, 2003).

### 4.2 Exam-oriented educational pressures

China’s exam-driven educational tradition also shapes learners’ motivation and emotions. Under this system, success is often measured by test scores rather than communicative competence, resulting in strong instrumental motivation but weak integrative engagement (Wang and Wen, 2002). The absence of standardized Spanish proficiency tests in China further creates uncertainty, frustration, and even emotional exhaustion (Li and Fan, 2016). Curricular practices typically prioritize grammar and translation, while listening and speaking remain underemphasized, producing a mismatch between written proficiency and oral fluency (Wang and Wen, 2002). Emotional support is also limited in such exam-focused contexts. Yet research suggests that strategies like positive reinforcement, collaborative learning, and emotion-focused instruction could substantially enhance learners’ confidence and resilience, though they are rarely implemented in practice (MacIntyre and Gregersen, 2012).

### 4.3 Social expectations and self-efficacy

Beyond cultural and educational contexts, social and familial pressures further shape Chinese students’ affective experiences in Spanish learning. In many families, foreign languages are regarded as cultural capital essential for academic advancement and future careers (Bourdieu, 1991). Such expectations can generate intense pressure, leading to anxiety and helplessness when students struggle to meet parental aspirations. Moreover, learners often report stronger self-efficacy in reading and writing than in speaking and listening, due to scarce opportunities for authentic communicative practice (Rui and Ji, 2017). Within the self-efficacy framework, mastery experiences, vicarious learning, and effective emotional regulation are regarded as central determinants of learners’ confidence and performance, yet opportunities for these mechanisms appear limited in Spanish classroom settings (Bandura, 1994). Consequently, students often equate mistakes with personal failure, fostering perfectionism, evaluation anxiety, and a “spiral of ability-related anxiety” that further undermines participation and confidence (MacIntyre and Gregersen, 2012).

## 5 Methodology

This study adopted a quantitative approach to systematically examine the psychological and emotional characteristics of Chinese students learning Spanish. Through a structured questionnaire, emotions were measured using culturally adapted and psychometrically validated scales of enjoyment, anxiety, and boredom. The questionnaire also assessed learners’ motivations, cultural influences, emotional states, and perceived learning outcomes.

### 5.1 Population and background

This study was carried out in November 2024 using a structured questionnaire distributed to a convenience sample of undergraduate students majoring in Spanish. The participants, ranging from freshmen to juniors, were enrolled in the Modern Spanish course at four Chinese universities, including two “211”^1^ universities and two ordinary universities. A total of 158 valid questionnaires were collected, among which 72 came from 211 universities and 86 from other universities. This sample was obtained through convenience sampling from only four universities, which may limit its representativeness. In terms of gender distribution, 32 participants were male and 126 were female. The average age of the respondents was 20.73 years (standard deviation = 0.81). All participants were in the early to middle stages of Spanish language acquisition and were receiving instruction in fundamental listening, speaking, reading, and writing skills. Prior to participation, all students provided informed consent. They were assured that their responses would remain anonymous and that the data collected would be used solely for research purposes.

Although students were recruited from four different universities, their Spanish courses followed the same national syllabus based on Modern Spanish. All participating institutions used the same examination paper during the data-collection, ensuring that academic performance was directly comparable across universities.

The study employed convenience sampling, and the resulting gender distribution (predominantly female) reflects the broader demographics of Spanish majors in China. This sampling approach, while practical and ecologically valid, may limit the generalizability of the findings to more gender-balanced populations.

To enable an in-depth analysis of learners’ emotional experiences and motivational orientations, the questionnaire was designed to gather not only basic demographic information but also multidimensional data on students’ learning motivation, cultural background, and psychological states. Specifically, the study focused on the following three key dimensions:

1. Motivational factors: This section explored students’ initial motivations for choosing Spanish as their major, as well as their ongoing sources of motivation throughout the learning process. The design incorporated interest-driven motivation (e.g., passion for the language or Hispanic culture), instrumental motivation (e.g., practical goals such as employment, studying abroad, or postgraduate education), and exam-oriented motivation (e.g., passive learning driven by the need to pass exams or fulfill course requirements). The objective was to determine whether different types of motivation were linked to distinct emotional patterns in language learning.
2. Cultural influences: This section examined the potential impact of traditional Chinese cultural values—such as face-saving, high-context communication styles, and collectivist tendencies—on students’ language learning experiences. It focused on behaviors such as avoiding classroom participation for fear of making mistakes, suppressing one’s natural communication style to align with perceived “normative” language standards, and feeling anxious about being judged for having a Chinese accent. This dimension aimed to uncover the intrinsic relationship between cultural values and language-related anxiety or communicative inhibition.
3. Learning stress: This section investigated whether students experienced emotional fluctuations—such as anxiety, boredom, or a diminished sense of accomplishment—during the learning process, and to what extent external pressures (e.g., family expectations, teacher evaluations, peer comparisons) affected their motivation and self-efficacy. By integrating these factors, the study sought to reveal how external socio-psychological pressures jointly influence students’ emotional responses and behavioral performance in Spanish language learning.

### 5.2 Research instruments

To assess students’ emotions and learning performance, the study used adapted, culturally contextualized psychometric scales measuring enjoyment, anxiety, and boredom, along with objective and self-reported outcomes. Guided by CVT, these emotions were selected because enjoyment reflects high perceived control and value, while anxiety and boredom stem from low control or value (Pekrun, 2006). This framework ensured theoretical consistency, and all scales showed good reliability and validity within the Chinese educational context. Each scale was translated from its original language into Chinese through a standard back-translation procedure and reviewed by bilingual experts in linguistics or education to ensure accuracy and cultural appropriateness of the items.

#### 5.2.1 Spanish learning pleasure scale

The Chinese version of the Spanish Learning Pleasure Scale was adapted from Li et al. (2018). It includes 11 items across three subdimensions: personal enjoyment, teacher-related enjoyment, and enjoyment of classroom atmosphere. Each item was rated on a 5-point Likert scale. The overall reliability was strong (Cronbach’s *α* = 0.896), with confirmatory factor indices indicating good model fit (CFI = 0.967, TLI = 0.951, RMSEA = 0.058).

#### 5.2.2 Spanish classroom anxiety scale

A reduced 8-item version of the Foreign Language Classroom Anxiety Inventory (Dewaele and MacIntyre, 2014) was adapted to focus on the Spanish classroom. Each item was rated on a 5-point Likert scale. The scale demonstrated high internal consistency (Cronbach’s α = 0.872) and solid structural validity (CFI = 0.961, RMSEA = 0.072).

#### 5.2.3 Spanish classroom boredom scale

This instrument was adapted from Li’s (2021) Foreign Language Classroom Boredom Subscale. Items were revised to reflect experiences in Spanish classes, particularly in exam-oriented contexts. The final scale showed excellent internal reliability (Cronbach’s α = 0.943) and strong construct validity (CFI = 0.971, RMSEA = 0.065).

#### 5.2.4 Learning outcomes measures

Learning outcomes were evaluated using two indicators: final exam performance and self-perceived Spanish learning effectiveness. The latter was measured with a 3-item scale adapted from Li et al. (2020), rated on a 10-point scale. Reliability was high (Cronbach’s α = 0.911). Exam scores were collected with informed consent and matched to the survey data.

#### 5.2.5 Cultural-psychological variables and embedded items

In addition to the established scales of enjoyment, anxiety, and boredom, this study incorporated cultural-psychological variables particularly relevant in the Chinese context. Previous research has emphasized that cultural values such as face-saving, high-context communication, and collectivist orientations strongly shape learners’ emotional experiences (Hall, 1976; Hu, 1944; Wen and Clément, 2003; Yu, 2016). Because most instruments in the field were developed in Western settings, they may not fully capture these dimensions. To address this limitation, culturally relevant items were embedded within the existing scales, ensuring that the measurement combined cross-culturally validated constructs with context-sensitive indicators of learners’ emotional experiences.

Examples of embedded items included:

*Face-saving concerns: “I feel embarrassed when I make mistakes in Spanish class.”*

*High-context communication: “I find it hard to speak up in class unless I feel fully prepared.”*

*Collectivist orientation: “I feel pressure to perform well because I do not want to let my group down.”*

These items were not designed as independent scales but rather as embedded indicators to illuminate how cultural-psychological traits intersect with emotional factors in the Spanish classroom. This approach preserved comparability with existing validated measures while also enhancing ecological validity in the Chinese learning context.

Although the embedded cultural-psychological items (face-saving, high-context communication, collectivist orientation) were not intended to function as independent scales, their internal consistency was examined to strengthen the credibility of these measures. The three-item cluster demonstrated acceptable internal consistency (Cronbach’s *α* = 0.74), and inter-item correlations ranged from 0.42 to 0.56, indicating moderate coherence among the items. These results support the reliability of the embedded indicators while maintaining their role as context-sensitive supplements rather than standalone constructs.

Although participants came from different academic years, their data were analyzed together because Spanish is typically introduced only at the university level in China, meaning learners across years share a similar starting point and trajectory. Separating them would have reduced statistical power, and preliminary comparisons showed no systematic differences across cohorts.

### 5.3 Data analysis methods

The reliability and construct validity of the four measurement instruments (including the enjoyment scale, anxiety scale, boredom scale, and perceived learning effectiveness scale) were examined using SPSS 29.0 and Mplus 8.3. Confirmatory Factor Analysis (CFA) was conducted in Mplus, and model modifications were made by allowing residual correlations where appropriate. The final models showed satisfactory fit indices (e.g., CFI > 0.95, TLI > 0.90, RMSEA < 0.08), indicating good structural validity of the scales. SPSS 29.0 was also used to conduct descriptive statistics, tests of normality, independent samples t-tests, Pearson correlation analyses, and stepwise regression analyses. Cronbach’s α values for all scales exceeded 0.85, indicating strong internal consistency reliability.

This study focused on three key aspects of statistical analysis. First, the interaction between emotional variables and cultural background was explored. Stepwise regression models included cultural variables such as institutional type, socioeconomic status, and family cultural capital to examine how these interact with emotions in shaping learning outcomes. For example, the analysis assessed whether face-related anxiety differs significantly between students at “211” universities and those at ordinary universities, or whether students from lower socioeconomic backgrounds are more vulnerable to pressure-related disruptions in academic self-efficacy. Second, group differences were analyzed. Through subgrouping by gender, institution type, and other variables, the study examined whether levels of enjoyment, anxiety, and boredom vary across groups and whether the relationship between these emotions and learning outcomes differs accordingly. Third, the interplay between emotional and psychological factors was investigated. Specifically, the analysis explored how emotional states interact with psychological traits such as self-efficacy and test-related stress, and how these jointly influence learning effectiveness.

Given the pronounced gender imbalance, any gender-based analyses were treated as exploratory and interpreted with caution. Although the gender distribution reflects the typical demographics of Spanish majors in China, we conducted additional robustness checks to minimize potential bias. Gender was included as a control variable in preliminary regression models, and the inclusion of this factor did not alter the significance or direction of the main predictors. This suggests that the core emotional patterns were not driven by gender-related effects. Group comparisons were conducted to provide descriptive insights rather than confirmatory evidence, acknowledging that the unbalanced sample may limit statistical power and external generalizability.

**Figure 1:**
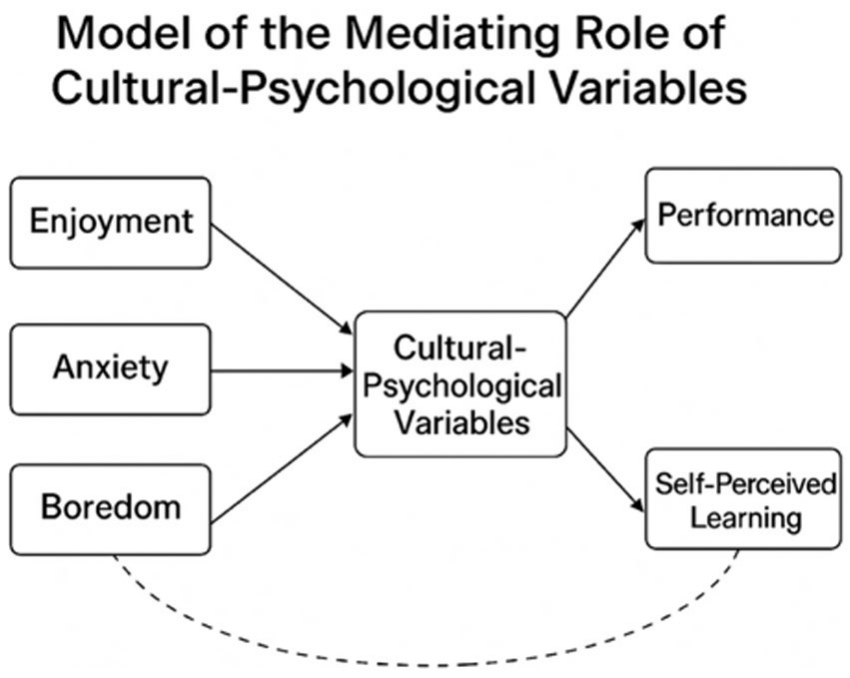
Model of the mediating role of cultural–psychological variables in Spanish language learning. This figure illustrates how emotional factors—enjoyment, anxiety, and boredom—affect Spanish learning outcomes through cultural–psychological mediators, which in turn influence both objective performance and students’ self-perceived learning. For transparency, the corresponding regression-based path coefficients are: Cultural → Anxiety (*β* = 0.31***), Cultural → Enjoyment (*β* = −0.22**), Cultural → Boredom (*β* = 0.18*), Cultural → Self-perceived effectiveness (*β* = −0.213**), and Cultural → Exam performance (*β* = −0.142*).

To deepen the analysis, this study introduces cultural-psychological variables as potential mediators between emotional states and learning outcomes. While values like face-saving and collectivism are known to influence language learning, their roles in emotion–performance dynamics remain underexplored. Drawing on control-value theory, we propose a conceptual model where enjoyment, anxiety, and boredom affect outcomes directly and indirectly via cultural variables. As illustrated in Figure 1, this model provides a culturally sensitive lens for understanding emotional processes in foreign language learning and contributes to context-specific frameworks in educational psychology.

While VIF statistics were not calculated in the current version, all predictors were conceptually distinct and exhibited low intercorrelations (|*r*| < 0.60), suggesting that multicollinearity was unlikely to bias the regression results. The regression analyses were conducted following standard assumption checks, and preliminary diagnostics confirmed stable parameter estimates across models.

## 6 Results

This study examined three core emotions—enjoyment, anxiety, and boredom—among Chinese learners of Spanish, focusing on their overall levels, interrelationships, and predictive roles in learning outcomes. Quantitative analyses, including descriptive statistics, correlations, and regression models, reveal how these emotions operate in classroom contexts shaped by both cultural background and exam-oriented pressures. The findings are interpreted with reference to the Control–Value Theory of Achievement Emotions (Pekrun, 2006) and the Broaden-and-Build Theory of Positive Emotions (Fredrickson, 2001, 2003), while also highlighting the specific features of Spanish learning as an L2 or L3 in China.

### 6.1 Overall emotional levels in Spanish classrooms

As shown in Table 1, enjoyment scores were significantly higher than both anxiety and boredom, suggesting that Spanish classrooms were, on the whole, emotionally positive environments. The relatively low variability in enjoyment indicated that most students consistently reported positive experiences, whereas larger individual differences were found for anxiety and boredom. These findings align with CVT, which posits that positive appraisals of control and value generate stable positive emotions (Pekrun, 2006).

**Table 1:** Descriptive statistics for key emotional and outcome variables.

Variable	Score Range	Mean	SD	Skewness	Kurtosis
Enjoyment	11–55	3.65	0.57	−0.31	1.03
Anxiety	8–40	3.12	0.81	0.47	0.18
Boredom	8–40	2.58	0.83	−0.26	0.25

Each emotional variable was rated on a 5-point Likert scale. *N* = 158. Self-perceived effectiveness was measured on a 10-point scale. Descriptive values for outcome variables may be inserted accordingly based on dataset.

Compared with research on English learning in China, where anxiety often dominates due to the high-stakes testing system (Dong et al., 2022; Horwitz, 2001), Spanish learners reported higher enjoyment and moderate anxiety. This pattern reflects the absence of large-scale standardized testing in Spanish and underscores the critical role of classroom climate and instructional design in shaping emotions when external pressures are weaker.

### 6.2 Correlations among emotions and learning outcomes

Correlation analyses (Table 2) revealed that enjoyment was negatively associated with both anxiety and boredom, while anxiety and boredom were positively correlated, suggesting the likelihood of co-occurrence. Enjoyment was positively correlated with both self-perceived learning effectiveness and exam scores, indicating its role in sustaining motivation and cognitive investment (Dewaele and MacIntyre, 2014). Anxiety and boredom, in contrast, correlated negatively with these outcomes.

**Table 2:** Pearson correlations among emotional variables and learning outcomes.

Variable	1	2	3	4	5
1. Enjoyment	—				
2. Anxiety	−0.294***	—			
3. Boredom	−0.502***	0.362***	—		
4. Exam performance	0.163*	−0.187*	−0.307***	—	
5. Self-perceived learning effectiveness	0.589***	−0.507***	−0.396***	0.451***	—

***p < 0.001, *p < 0.05. *N* = 158. All correlations are two-tailed. Variables 1–3 measured emotional states; Variables 4–5 represent objective and subjective outcomes.

### 6.3 Predictive power of emotions on learning outcomes

Regression results (Table 3) showed that anxiety was the strongest negative predictor of exam performance (*β* = −0.384, *p* < 0.001), while enjoyment and boredom were excluded. This confirms CVT’s prediction that negative appraisals of control undermine achievement in test situations. However, in predicting self-perceived learning, enjoyment was the strongest positive predictor (*β* = 0.436, *p* < 0.001), while boredom had a significant negative effect (*β* = −0.278, p < 0.001). Anxiety was excluded in this model.

**Table 3:** Stepwise regression predicting learning outcomes from emotional variables.

Dependent variable	Predictor	*β* (Standardized)	95% CI for *β*	*t*	*p*	Partial *η*^2^
Exam performance	Anxiety	−0.384	[−0.493, −0.275]	−7.615	<0.001	0.147
	(Enjoyment, Boredom)	(Excluded)	—	—	—	—
Self-perceived learning effectiveness	Enjoyment	0.436	[0.346, 0.525]	8.976	<0.001	0.209
	Boredom	−0.278	[−0.359, −0.197]	−5.710	<0.001	0.101
	Anxiety	(Excluded)	—	—	—	—

*β* = standardized regression coefficient. Confidence intervals are bootstrapped (5,000 samples). Partial *η*^2^ indicates effect size per predictor.

This contrast demonstrates the dual role of emotions in Spanish learning: anxiety directly undermines exam performance, consistent with findings in English learning (MacIntyre and Gregersen, 2012), while enjoyment and boredom shape learners’ subjective engagement with the language. Unlike English, where large-scale exams intensify the role of anxiety, Spanish learning relies more heavily on classroom dynamics, making enjoyment and boredom especially salient predictors of perceived success.

### 6.4 Associations between cultural-psychological variables and emotions

To examine the role of cultural-psychological characteristics in Spanish learning, three embedded items—face-saving, high-context communication, and collectivist orientation—were combined into a cultural-psychological cluster with acceptable reliability (*α* = 0.74; inter-item *r* = 0.42–0.56). As shown in Table 4, this cluster correlated significantly with the three emotions: it was positively associated with anxiety (*r* = 0.31, *p* < 0.001) and weakly with boredom (*r* = 0.18, *p* = 0.027), while showing a negative association with enjoyment (*r* = −0.22, *p* = 0.009). These patterns indicate that culturally shaped communicative inhibition and evaluative concerns heighten emotional vulnerability and reduce positive emotional engagement.

**Table 4:** Correlations between cultural-psychological cluster and emotional variables (*N* = 158).

Variable	FLE	FLA	FLB
Cultural-psychological cluster	−0.22*	0.31***	0.18*

FLE, enjoyment; FLA, anxiety; FLB, boredom. ***p < 0.001, *p < 0.05.

Regression analyses further showed that cultural-psychological traits explained additional variance beyond the core emotions. After controlling for anxiety, they exerted a small but significant negative effect on exam performance (*β* = −0.142, *p* = 0.042) and a moderate negative effect on self-perceived effectiveness (*β* = −0.213, *p* = 0.008), suggesting that face-saving concerns and communication restraint lower learners’ confidence and perceived success.

To increase transparency in the conceptual model, the corresponding path coefficients were incorporated into Figure 1 (Cultural → Anxiety: *β* = 0.31***; Cultural → Enjoyment: *β* = −0.22**; Cultural → Boredom: *β* = 0.18*; Cultural → Self-perceived effectiveness: *β* = −0.213**; Cultural → Exam performance: *β* = −0.142*). Overall, cultural-psychological traits—particularly face-saving and high-context communication—were significantly linked to emotional experiences and learning outcomes, supporting the role of cultural values in shaping Spanish learning emotions and complementing the broader Control–Value Theory framework.

### 6.5 Summary of results

Overall, the results show that Chinese learners of Spanish experience relatively high enjoyment, moderate anxiety, and variable boredom. These emotions interact in predictable ways—enjoyment broadens engagement, while anxiety and boredom constrain it—but the Spanish learning context highlights a unique pattern: in the absence of systemic testing, enjoyment and boredom become particularly decisive in shaping learners’ self-perceptions, while anxiety remains the primary barrier to exam outcomes. Cultural-psychological tendencies, especially face-saving and high-context communication, were also meaningfully linked to these emotions: they predicted higher anxiety and boredom, lower enjoyment, and modest but significant decreases in exam performance and self-perceived effectiveness. Together, these findings highlight the need to consider both L2/L3 context-specific emotional patterns and culturally shaped communication norms, rather than assuming that results from English-learning environments generalize automatically to Spanish.

## 7 Discussion

Emotions are key to foreign language learning, influencing not only learners’ motivation and classroom performance but also reflecting deeper cultural and psychological dimensions. This study investigates the emotional experiences of Chinese university students learning Spanish, focusing on the interactions between three core emotions—enjoyment, anxiety, and boredom. By analyzing these emotions within the context of Chinese sociocultural and educational frameworks, this study reveals how emotions shape learning outcomes and provides practical, context-specific pedagogical recommendations. Through this process, we gain deeper insights into how emotions impact Spanish language learning in China, supporting a more human-centered approach to foreign language instruction.

### 7.1 Emotional characteristics in Spanish language learning among Chinese undergraduates

The results indicate that Chinese learners of Spanish typically experience a blend of enjoyment and anxiety, along with moderate levels of boredom. This emotional coexistence reflects the unique challenges faced by learners of a less commonly taught language like Spanish. The novelty and phonetic appeal of Spanish, along with its rich cultural connections to Latin America and Spain, stimulate interest and enjoyment. However, the unfamiliarity of the language compared to English, its complex grammar, and the lack of real-world application contribute to emotional strain, which triggers anxiety, frustration, and learning fatigue.

While some students initially find Spanish attractive due to its transparent pronunciation and early progress, the increasing complexity of the curriculum—especially in vocabulary and verb conjugation—can lead to emotional overwhelm. Those motivated intrinsically by career goals or cultural interest tend to experience more enjoyment and sustained engagement. On the other hand, extrinsically motivated students, focused on fulfilling academic requirements, report higher levels of frustration and disengagement, transitioning from curiosity to boredom and fatigue.

This emotional shift can undermine persistence and ultimately affect academic performance. Therefore, programs should focus on sustaining intrinsic motivation through meaningful engagement, so students do not view language learning as merely a tool to complete tasks but rather as a valuable and enjoyable skill for personal growth.

### 7.2 Interrelations among the three emotions

This study explored the relationships between enjoyment, anxiety, and boredom in Spanish learning, revealing significant emotional dynamics. Negative correlations were found between enjoyment and both anxiety and boredom, while anxiety and boredom were positively correlated. These findings align with Dewaele and MacIntyre’s (2014) “coexistence theory” of foreign language enjoyment and anxiety, suggesting that positive emotions buffer the negative effects of anxiety and boredom.

Enjoyment in Spanish learning is driven not only by the language’s intrinsic qualities—such as its phonetic charm and cultural richness—but also by the quality of classroom interactions and the teaching approach. A supportive, interactive classroom environment can significantly reduce anxiety and boredom. However, these effects are shaped by cultural factors. In Chinese culture, silence is often viewed as a sign of respect and thoughtfulness. In contrast, Spanish classrooms encourage fluency and spontaneity, where silence can be misinterpreted as disinterest or incompetence, leading to heightened anxiety.

Additionally, tasks such as speaking or role-playing often trigger “perfectionist silence,” where students hesitate to speak unless they feel their response is near-perfect. This fear can lead to boredom—not because the class is dull, but because students feel emotionally withdrawn and unable to engage fully. This emotional withdrawal creates a cycle where anxiety and boredom reinforce each other, often leading to avoidance behaviors, such as zoning out or passive disengagement, which in turn slow down language progress.

These findings indicate that emotional dynamics in Spanish learning are not merely statistical correlations but are deeply embedded in culturally specific psychological mechanisms. Teachers need to develop emotional sensitivity and cultural adaptability to detect students’ emotional states in real time. Through appropriate feedback, scaffolding, and task design, teachers can help students overcome emotional withdrawal, fostering enjoyment and active participation.

### 7.3 Emotional predictors of learning outcomes

The findings highlight the complementary roles of CVT and BBT. CVT, as the primary framework, explains how enjoyment, anxiety, and boredom arise from control–value appraisals and shape immediate learning outcomes (Pekrun, 2006). BBT adds a longer-term perspective, showing how enjoyment broadens engagement and builds enduring resources such as resilience and confidence (Fredrickson, 2001). Together, these theories clarify both the short-term and long-term effects of emotions in learning, explaining why enjoyment promotes growth while anxiety and boredom impede it.

This study found that Foreign Language Anxiety (FLA) significantly predicts students’ exam performance in the specific context of Chinese university Spanish written exams, while enjoyment and boredom are more strongly associated with self-reported learning effectiveness. These findings align with the Control–Value Theory of Achievement Emotions (Pekrun, 2006), which posits that students’ emotional experiences are shaped by their appraisals of both control (perceived ability to manage learning tasks) and value (importance of the tasks).

Anxiety plays a complex role in China’s exam-driven educational system. While moderate anxiety can enhance focus and concentration, excessive anxiety disrupts cognitive processing and impairs performance. In contrast, enjoyment contributes to deeper learning: students who report higher levels of enjoyment are more likely to exhibit internalized motivation and a positive attitude toward course content. Boredom, on the other hand, significantly reduces engagement and leads to negative evaluations of the learning experience.

In China, where exam results are often the sole indicator of success, students’ emotional engagement and well-being are frequently overlooked. This disconnect between objective performance and subjective experience suggests a need to shift how language learning outcomes are evaluated. Emotional engagement should be considered as an integral part of assessing student success, alongside traditional measures like exam scores. This study identified a specific link between anxiety and written exam performance in Chinese university Spanish courses emphasizing grammar and writing. Oral assessments were not examined, leaving their effects unaddressed. Accordingly, the findings should be interpreted within this context, and future research should compare emotional impacts across different assessment formats and learning environments.

### 7.4 Cultural and emotional intersections in language learning

These results further suggest that cultural-psychological traits exert meaningful influences on emotional experiences in Spanish learning, consistent with previous literature (Hu, 1944; Hall, 1976; Wen and Clément, 2003; Yu, 2016). The emotional challenges faced by Chinese students learning Spanish are notably more pronounced than those encountered in English language learning. Spanish, unlike English, does not hold the same broad societal and professional utility in China, leading to what can be described as “motivational drift.” Without clear reinforcement from external sources, students struggle to sustain motivation, experiencing emotional fatigue and inefficacy. While English is widely viewed as a global lingua franca that provides strong career advantages, Spanish lacks comparable societal demand and academic support, which reduces learners’ exposure and opportunities for authentic language use outside the classroom.

Additionally, Chinese students are often more risk-averse in communication, fearing mistakes and prioritizing accuracy over fluency (Biggs, 1996; Hu, 1944). This accuracy orientation fosters perfectionism and creates psychological obstacles that lower students’ willingness to engage in spontaneous speech. Cultural norms related to face preservation and teacher authority further reinforce silence and withdrawal (Wen and Clément, 2003), while heightened sensitivity to peer judgment exacerbates anxiety in small-group Spanish classes. Meta-analytic evidence shows that such anxiety and fear of negative evaluation can have a stronger impact on second-language achievement than linguistic knowledge alone (Teimouri et al., 2019).

From a control–value perspective, exam-oriented pressures undermine students’ perceived control over learning: high-stakes tests make success feel less within their control, thereby heightening anxiety. Likewise, face-saving norms amplify the perceived value of performance (and the cost of mistakes), intensifying pressure and diminishing enjoyment. These cultural factors shape the emotional experiences observed among Chinese learners of Spanish.

To address these challenges, Spanish instruction in China should be student-centered and supportive. Teachers should normalize errors, emphasize communicative competence and cultural context over high-stakes testing, and foster confidence and emotional resilience in their students.

## 8 Conclusion

This study examined the emotional dynamics of Chinese university students learning Spanish, focusing on the interplay of enjoyment, anxiety, and boredom. Results showed that enjoyment was the most prevalent and constructive emotion, positively associated with learners’ perceived effectiveness, while anxiety significantly predicted lower exam performance. Boredom, though often overlooked, consistently undermined engagement and motivation. Together, these findings reaffirm that emotions are central—not peripheral—to foreign language learning success.

The results highlight the value of integrating CVT and BBT. CVT served as the primary framework, explaining how enjoyment, anxiety, and boredom arise from students’ appraisals of control and value and shape learning outcomes. BBT complemented this by clarifying the benefits of enjoyment, showing how it broadens engagement and builds long-term resources (Fredrickson, 2001). Together, they provide a more comprehensive account of why enjoyment enhanced learning while anxiety and boredom hindered it. Importantly, the study demonstrates that these emotions are culturally embedded. In China, face-saving norms, high-context communication, and exam-oriented practices strongly influence how learners experience and regulate their emotions. Spanish learning, therefore, cannot be understood in isolation from the sociocultural framework in which it takes place.

A key contribution of this study lies in clarifying the distinctiveness of Spanish learning in the Chinese context. Unlike English, which benefits from societal exposure, high instrumental value, and strong institutional support, Spanish occupies a more peripheral position. Learners depend heavily on classroom interactions, making enjoyment, anxiety, and boredom especially salient in shaping outcomes. In contrast to English, where standardized testing often amplifies anxiety, Spanish learning is more strongly affected by intrinsic motivation and classroom climate. While some of these dynamics may also be found in other less commonly taught languages, such as French or Russian, Spanish offers a particularly compelling case due to its global rise but still limited institutional support in China.

Based on these findings, we recommend that Spanish instruction should prioritize emotionally supportive and communicative environments. Classrooms where mistakes are normalized, peer collaboration is encouraged, and risk-taking is valued can help sustain enjoyment, reduce anxiety, and prevent boredom.

Despite these contributions, several limitations should be acknowledged. While the study relied on convenience sampling and included a gender imbalance typical of Spanish majors in China, these limitations should be considered when interpreting the results. Additionally, the cross-sectional design provides only a static snapshot of learners’ emotions; thus, references to ‘emotional dynamics’ here denote the concurrent interplay of enjoyment, anxiety, and boredom at one point in time rather than changes over time. Future research should employ stratified sampling and moderation analyses to test whether gender influences the emotional pathways identified here.

Finally, the broader significance of this study extends beyond Spanish. It contributes to the cross-linguistic understanding of how emotions shape language learning, particularly in contexts where societal reinforcement is limited. By highlighting the unique position of Spanish as an L2/L3 in China, this study underscores the need for more human-centered, culturally responsive, and emotionally sustainable approaches to foreign language education.

## Data Availability

The datasets presented in this study are not readily available because they contain sensitive information related to students’ educational experiences and personal responses. Participants did not provide consent for their raw data to be publicly shared, and institutional ethical requirements restrict open dissemination. Requests to access the datasets should be directed to YL (yitoliu@ruc.edu.cn).

## Footnotes

1 “211 universities” are top-tier Chinese institutions receiving prioritized government support since 1995 under the national project 211 initiative.
